# Characterization of Urban Subway Microenvironment Exposure—A Case of Nanjing in China

**DOI:** 10.3390/ijerph16040625

**Published:** 2019-02-20

**Authors:** Peng Mao, Jie Li, Lilin Xiong, Rubing Wang, Xiang Wang, Yongtao Tan, Hongyang Li

**Affiliations:** 1 Department of Construction Management, College of Civil Engineering, Nanjing Forestry University, Nanjing 210037, China; maopeng@njfu.edu.cn (P.M.); wangrubinghappy@163.com (R.W.); yzwx97@163.com (X.W.); 2 Department of Construction Management and Real Estate, School of Civil Engineering, Shenzhen University, Shenzhen 518000, China; lijie20181@email.szu.edu.cn; 3 Department of Environmental Health, Nanjing Municipal Center for Disease Control and Prevention, Nanjing 210037, China; hzxionglilin@163.com; 4 Department of Building and Real Estate, The Hong Kong Polytechnic University, Hung Hom, Kowloon, Hong Kong 999077, China; yongtao.tan@polyu.edu.hk; 5 Department of Construction Management, School of Civil Engineering and Transportation, South China University of Technology, Guangzhou 510641, China

**Keywords:** urban subway, microenvironment exposure, field sampling, protective strategies, standard update

## Abstract

Environmental quality in public rail transit has recently raised great concern, with more attention paid to underground subway microenvironment. This research aimed to provide guidance for healthy urban subway microenvironments (sub-MEs) according to comprehensive micro-environmental categories, including thermal environment, air quality, lighting environment, and acoustic environment from both practical and regulation perspectives. Field sampling experiments were conducted in Nanjing Metro Line X (NMLX). Descriptive analysis, correlation analysis and one-way analysis of variance were used to investigate the status quo of urban sub-MEs. A paired samples t-test was then performed to compare among subway station halls, platforms, and in-cabin trains based on integrated sub-MEs. Results show that relative humidity, air velocity, respirable particulate matter (PM_10_) concentration, and illuminance dissatisfy the requirements in relevant national standards. Significant difference was observed in lighting environment between station hall and platform. It was detected platforms are warmer and more polluted than train cabins. Additionally, subway trains generate main noise on platform which is much louder when leaving than arriving. Protective strategies for sub-ME improvement as well as principles for updating standards were proposed from a proactive point of view. The findings are beneficial for moving towards healthy urban sub-MEs and more sustainable operation of subway systems.

## 1. Introduction

Public rail transit witnesses a remarkable leap forward with the rapid growth of urbanization in different countries/regions all over the world [1]. The underground subway, due to its efficiency, safety, and large-scaled passenger capacity, has gradually become one of the most prevalent commuting modes in rail transit systems [2,3]. According to the Statistics and Analysis Report of Urban Rail Transit [4], the total passenger volume of rail transit nationwide had increased to 18.48 billion and there are 3884 km subway lines, at the proportion of 77.2% of urban rail transits, operated in China by the end of 2017.

Despite the convenience brought by subway, one serious environmental disadvantage was observed that different air pollutants may accumulate in such confined space of train cabins where the commuters typically spend around 75% of their commuting time [5,6]. In recent years, underground air quality has been more concerned as the concentrations of air pollutants indoors are higher than the ambient levels even in some matured urban subway systems [7,8,9,10]. Furthermore, much of inhalable particulate matters on subway platforms is actually produced underground—quite different from that breathed outdoors [11].

Previous studies have shown that the exposure to underground transport system exacerbates adverse health effects of passengers, such as acute changes in heart rates, pulmonary dysfunction, cardiovascular diseases, etc., [12,13,14]. Besides, Jung et al. [15] confirmed that heavy metals, the major ingredients of particulate matter (abbreviation PM thereafter) in subway air, pose great threats to human health.

Different understandings existed regarding the microenvironment of subways in prior studies. For instance, it comprises the commuting mode and relevant boarding/alighting facilities (e.g., station platforms) [16]. Considering the fact that underground transport system exposes urban population to various sources of air pollution daily, subway commuting together with home and workplace constitute three main microenvironments compared with the ambient environment [17]. In the current research, urban subway microenvironments (sub-MEs, including station halls, platforms, and train cabins), composed of confined spaces, are a special category of indoor microenvironment [18,19]. The stable microenvironment indoors is characterized by fresh air flowing, natural lighting, no noise disturbing, and comfortable thermal conditions [20].

Exposure to urban subway microenvironments has drawn much concern since they are relatively enclosed and poorly ventilated [21]. Studies focusing on field monitoring in subways have increased extensively. Air pollutants were primarily observed as ascribed to a wide range of sources, such as traffic emissions, wheels, catenaries, brake pads, and infiltration from out-stations [22]. The volatile organic compounds (VOCs) concentration inside subway cabins was ranked the second among different transport modes [23]. Total volatile organic compounds (TVOCs) levels, bacterial levels, and PM_10_ concentrations of underground were detected exceeding the stipulated standards as revealed in the research of Chen et al. [24]. The subway PM concentrations were almost 20% greater than those in ambient air [25]. Furthermore, studies have been carried out focusing on underground inhalable particles in subway systems of many other countries and cities like Finland, Mexico, Taipei, and Barcelona [11,26,27,28]. A prevailing conclusion has been reached that commuters, on the daily basis, are exposed to subway microenvironments full of heavy air pollutants. However, most of the existing studies defined sub-MEs from only one perspective of indoor air quality (particularly PM exposure in subway systems), an integrated consideration of overall micro-environmental categories is still lacking. Simultaneously, many scholars, especially in Europe, paid much attention on physicochemical compositions and their potential health implications from exposure to air pollutants in sub-MEs. Nawrot et al. [29] suggested traffic-related air pollution could be a vital trigger of heart attacks than drug abuse for general population. Cheng and Lin [27] proposed that the common subway airborne particles, including heavy metals, e.g., Ferrum (Fe), Manganese (Mn), Chromium (Cr), Nickel (Ni), and Cuprum (Cu), can negatively affect human bodies. Karlsson et al. [30] pointed out that geno-toxicity of underground particles is seven times higher than that in the roadway, leading to increased oxidative pressure in lung tissues. Besides, it is evidently that in subway air, of which hydrocarbons, fungus and bacteria, metallic elements, and some additional toxic compositions on different concentration levels would result in different health risks, such as oxidative stress in lung tissues, excess cancer mortality, and some other increased diseases [26,31,32,33]. Nevertheless, the focus of these studies is directed to post-health effects of air pollutants underground on commuters, failing to prevent environmental issues before they emerge. Hence, it is of great necessity to propose protective strategies according to various microenvironment categories to mitigate negative impacts in sub-MEs in a proactive manner.

In practice, though China have promulgated some standards or norms related to indoor air quality or urban subways, the relevant environmental indicators as well as their standardized thresholds can hardly match with the variability of the sub-ME nowadays. Ministry of Environmental Protection of the People’s Republic of China has proposed the revised Ambient air quality standards (GB3095-2012) in August 2018. However, such a standard only concentrates on ambient air, and no revisions are underlined in terms of indicators and thresholds. To effectively improve subway microenvironments and therefore ensure public health, it is essential to formulate an updated and targeted standard on sub-ME integration. 

Nanjing, one of the most prosperous and densely populated cities in China, is a pioneer where the urban subway systems are open across on county levels, covering all eleven municipal districts. There are 10 subway lines and 174 subway stations in Nanjing at present. The total length of lines has reached 364.3 km, and another fifteen subway lines (1011.2 km) are expected step into operation in 2030 according to Nanjing City Urban Master Plan (2007–2030). It is estimated that the Nanjing subway system carries over 34 million passengers per day on average, with the peak up to 40.2 million [34]. As a result, underground environmental quality in sub-MEs undoubtedly becomes a pervasive issue pertaining to public health in Nanjing. Generally, the previous findings suggest exposure of commuters is individualized due to the heterogeneity of transport system in each city. Tailored studies are needed by taking into account the field scenarios so that more accurate results could be produced regarding local transport-related exposure [35].

The specific objectives of this research are therefore set, as to: (1) investigate the current situations of urban sub-MEs regarding full micro-environmental categories with field measurement in the city of Nanjing; (2) characterize disparities of urban sub-MEs among subway station halls, subway platforms, and in-cabin trains; (3) propose protective strategies for urban sub-MEs improvement according to different micro-environmental categories; and (4) develop corresponding principles for standards or norms regarding urban subway microenvironments. The research explores broad thoughts of operation management to improve microenvironment quality in urban subway systems. The findings are expected to help step towards healthy urban sub-MEs from a proactive view on practice and regulation levels and facilitate sustainable operation of subways.

## 2. Research Process and Methods

### 2.1. Sampling Area and Sites

Opened in 2015, Nanjing Metro Line X (NMLX) of 44.9 km in length and with forty-six Model-A trains (276 carriages in total) in service, was selected as the sampling case. It starts from the Linchang viaduct station on the north and ends at the East Mozhou Road station on the south—passing several important transportation hubs, downtown districts, and main residential areas, e.g., Nanjing Railway Stations, Jiangbei Pukou, Jiangnan Gulou, Xuanwu, etc. Twenty-nine stations established are all underground with only one exception. Among these stations, there are five interchange ones in operation, and another eight are still under construction or planning, showing a good function of transfer interface.

**Figure 1:**
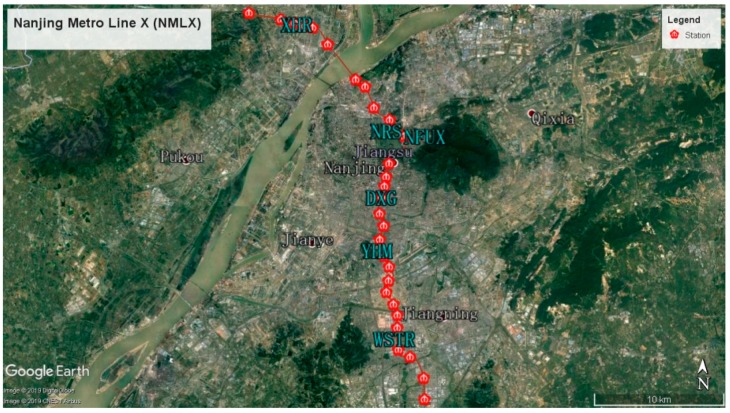
Sampling Area of Line X of the Nanjing Metro.

To ensure the representativeness and generality of the research findings necessitates the selection of both typical suburban stations and downtown stations as monitoring sites. Another two interchange stations were also targeted based on the consideration of the functional characteristics. Simultaneously, all of the chosen stations are located underground, which are relatively enclosed and poorly ventilated. A random sampling approach was then applied and five trains (thirty carriages) chosen with official codes as 033034, 049050, 065066, 077078, and 083084 respectively. Details of the monitoring sites and conditions are shown in Table 1 and the layout of these selected stations illustrated in Figure 1.

### 2.2. Selection of Indicators and Criteria

Many countries have established their own national standards of ambient air quality following the WHO Air Quality Guidelines, e.g., the Ambient air quality standards of China (GB3095-2012) [36,37,38]. Nevertheless, there is great vacancy in broad legally binding force on indoor air quality standards, let alone that on cabins of vehicles [39]. Chinese government plays a primary role with regard to this issue. To date, relevant standards or norms associated with indoor environments or urban subways have been promulgated nationwide, such as the Hygienic standard for waiting room of public transit means (GB9672-1996), the Code for design of metro (GB50157-2013), the Code for indoor environmental pollution control of civil building engineering (GB50325-2010), and the Urban rail transit lighting (GB16275-2008). Sampling indicators were then identified based on sub-ME categories in literature retrieval and the current national standards above [19,24,31]. For experimental quality control, the most rigorous value was selected as the final criterion of indicator if the standard limits appeared different with each other (Table 2).

### 2.3. Sampling Instruments

To avoid measurement errors, professional instruments were used to monitor sampling indicators. Digital thermometer, model Testo 625 (TESTO Instrument Co., Ltd., Shanghai, China), was able to measure temperature and relative humidity (RH) with an accuracy of ±0.5 °C and ±2.5% RH. Air velocity was monitored using an anemometer, model 9565-X (TSI, TN, USA) with the top value up to 10.16 m s^−1^. By using a set of portable CO_2_ analyzer, i.e., model GXH-3010E (Institute of Beijing HUAYUN Analytical Instrument Co., Ltd., Beijing, China), CO analyzer, model GXH-3011A (Institute of Beijing HUAYUN Analytical Instrument Co., Ltd., Beijing, China) and laser dust meter, model LD-3C(B) (Beijing Greenwood Innovative Digital Technology Co. Ltd., Beijing, China), we measured real-time CO_2_, CO, and PM_10_ mass concentrations underground. As for two more indicators regarding (1) air quality, TVOC was monitored using multifunctional portable environmental quality inspection system instrument (Gray Wolf Sensing Solutions, LLC, Shelton, CT, USA) within 10% accuracy; and (2) airborne bacteria were sampled by numerous transparent petri dishes which were treated with high temperature steam produced by autoclave sterilizer. An extra 240-litre incubator, model BD240 (BINDER GmbH Headquarters, Tuttlingen, Deutschland), was then used for bacterial culture after the field sampling. The colony counting was conducted after 48-hour-culture at 37 °C. In addition, an illumination photometer, model TES-1332A (TES Electrical Electronic Corp., Taipei, China) was used for illuminance samplings in subway lighting environment while a noise dosimeter, model AWA5610D (Hangzhou Aihua Instruments Co. Ltd., Hangzhou, China) measure noise levels in its acoustic environment. Before field sampling, the instruments were well prepared in their exclusive containers and each kind of instruments was carried by an investigator, who had been rigorously trained for instrumental calibrations and operations.

### 2.4. Measurements and Quality Control

**Figure 2:**
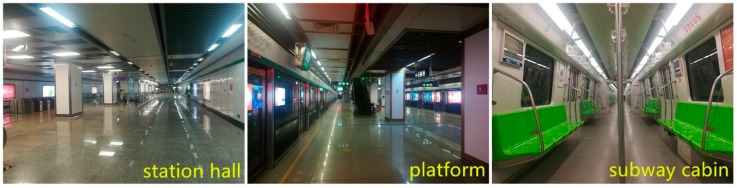
Sampling Sites of Nanjing Metro Line X (NMLX).

The field samplings in three categories of sub-MEs (i.e., thermal environment, air quality, and lighting environment) were conducted in station halls, platforms and subway cabins (Figure 2). This research follows the current standard of subway acoustic environment, viz. the Acoustical Requirement and Measurement on Station Platform of Urban Rail Transit (GB14227-2006) and concentrates on platform situation when monitoring acoustic environment. Besides, the noises on platforms are found in the pilot study that they are commonly composed of e.g., subway trains arriving or leaving, out screen broadcasting, talking noises, and even shoes clattering of passengers.

The whole process of sampling point selection and measurements in sub-MEs are rigorously in line with the latest national standards, including the Examination methods for public places—Part 1: physical parameters (GB/T18204.1-2013), the Examination methods for public places—Part 2: Chemical pollutant (GB/T18204.2-2014), the Examination methods for public places scrutiny—Part 3: Airborne microorganism (GB/T18204.3-2013), the Examination methods for public places—Part 6: Technical specifications of health monitoring (GB/T18204.6-2013), and the Acoustical requirement and measurement on station platform of urban transit (GB14227-2006), where the measurements are incompliance with those of the limit values selected.

**Figure 3:**
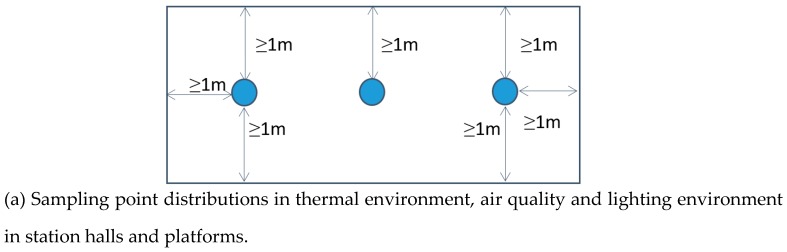

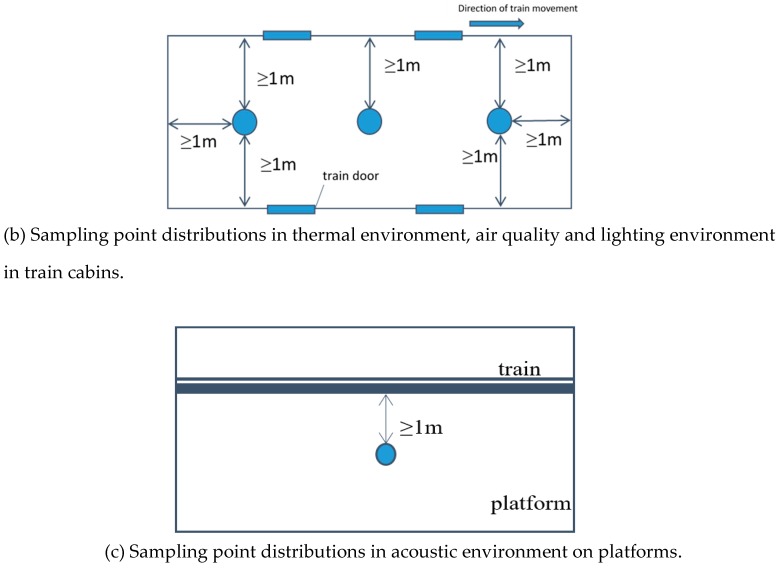
Distributions of sampling points in subway station halls, platforms and cabins.

According to the standards above, at least three sampling points are required with an air monitoring area over 1000 m^2^. Hence, the points based on indoor depth were selected, and those of station halls and platforms were aligned vertically. Same points were distributed with regard to illuminance monitored, while they were separately designed for acoustic measurement. When the noise was monitored, it was positioned at the middle of the platform with one meter away from screen doors. As for sub-MEs in cabins, we experimented in two head carriages as well as a body carriage of each train, where sampling points were similarly located to those in stations halls or platforms. Three points were then selected based on the length of carriage. Detailed distributions of the sampling points are shown in Figure 3.

According to Figure 3, we conducted the sampling in point coding order, and monitored ME-indicators simultaneously at each point. Subway cabins were monitored in time intervals before we arrived at the next sampling location. Since the experiments were conducted in ventilation season, it was ventilation systems rather than cooling systems of air conditioning that were operated at the time of sampling. Besides, no air curtain was opened at the entrance of each station hall. All platforms were equipped with screen doors.

Measurement at each point was tested via collocated sampling. Side-by-side comparisons were made twice for samplings in sub-ME of thermal environment, air quality, and lighting environment. Following the standard operation procedures in the Acoustical Requirement and Measurement on Station Platform of Urban Rail Transit (GB14227-2006), noise was monitored individually regarding acoustic environment. It was sampled ten separate times when trains were arriving and leaving platforms. Moreover, equivalent consecutive sound level A (LAeq) under the circumstance of no trains passing was monitored as fundamental noise levels on platforms. Specifically, it was monitored five times when screen broadcast was on, and another five with broadcast off. Mean value was finally calculated for sampling measurement of each sub-ME indicator.

The sampling experiment was conducted without impact on daily passengers commuting in NMLX. It was assisted by specialists from Nanjing Municipal Center for Disease Control and Prevention during the whole sampling process. We collected all air samples at the height of breathing zone (0.5–1.5 m) [40]. Similarly, when illuminance and noise levels were monitored, the sampling inlets of instruments were located at roughly 1.5 m above the ground. For validity of air samples, instruments were kept off the zones with strong wind, such as ventilation shafts, air outlets, and screen door-sides, and kept at least one meter away from walls or carriage bodies. Moments of passengers on and off were shunned in terms of cabin samplings. Furthermore, the sampling time selected was reasonable, as it avoided rainy days and daily rush hours (7.00–9.00 a.m. and 17.00–19.00 p.m.). All instruments were seriously zero-calibrated before each measurement carried out at sampling locations. Monitors at one point were set to around 6-s sampling intervals [37]. The data obtained were timely logged by trained lab assistants. All measurements of sub-MEs were completed on the same day in order to avoid ambient influence on the daily basis.

### 2.5. Data Analysis

All the data shown in mean ± standard deviation (SD), were measured within their individual 95% confidence interval. By using SPSS version 22.0 (IBM, Armonk, NY, USA), we firstly used descriptive statistics to illustrate the current situations of urban sub-MEs in typical station halls, platforms, and in-cabin trains. Correlation analysis via Spearman correlation coefficient was adopted to explore the correlations of various sub-ME monitoring indicators and significance was defined as *p* < 0.01. One-way analysis of variance (ANOVA) was used to make sub-ME comparisons according to station types, i.e., suburban station, interchange station, and downtown station. We then performed a paired samples t-test to test differences in sub-MEs of station halls versus platforms, platforms versus trains, and various acoustic environments on platforms. Significance was defined as *p* < 0.05.

## 3. Results

### 3.1. Status quo of sub-MEs in NMLX

Table 3 illustrates the current sub-ME situations of different stations in NMLX. Among the six stations selected, indicators, with values satisfying the thresholds set in relevant standards or norms in China, are temperature, CO_2_ concentration, CO concentration, TVOC concentration, total count of airborne bacteria, and noise levels. Relative humidity (RH) was found exceeding the limit easiest in subway thermal environment. Over 50.0% of station halls and platforms experienced high RH out of limits, with the top of 73.7% (SD = 5.5%) in station hall of NFUX station. Regarding air quality underground, it is worth noticing that beyond-standard PM_10_ concentration simultaneously occurred in station halls as well as platforms of YHM station and WSTR station. Meanwhile, we found PM_10_ on platforms seemed much serious than that in station halls. PM_10_ concentration on YHM platform (Mean = 0.336 mg m^−3^, SD = 0.035 mg m^−3^) was monitored around 1.3 times of that in its station hall (Mean = 0.259 mg m^−3^, SD = 0.023 mg m^−3^). Similarly, PM_10_ concentration on WSTR platform (Mean = 0.362 mg m^−3^, SD=0.113 mg m^−3^) was approx. 1.1 times higher than that in the upper station hall (Mean = 0.338 mg m^−3^, SD = 0.198 mg m^−3^). Substandard lighting environment was commonly found in both station halls and platforms. More than 80% of stations were monitored, whose illuminance of station halls was lower than that recommended in the standards. Station hall of YHM station tolerated the most terrible rayless light among the six, with average illuminance of 59.2 lx (SD = 57.2 lx). Besides, we sampled poor illuminance, which was below the recommended value, on around half of platforms, including NFUX, YHM, and WSTR station. The most uncomfortable lighting environment on platforms existed in WSTR station, the average illuminance of which was measured only 35.0 lx (SD = 3.7 lx). It was not detected quite noisy hubbub on each platform of subway stations. Average of noise levels under three conditions, viz. no trains, train arriving, and train leaving, was no more than the lowest standard limit of 70 dB(A). When compared with station halls and platforms, better sub-MEs were recorded in cabins of carriages, where all ME-indicators measured conformed to the standard except RH (Mean = 70.2%, SD = 0.7%) in their thermal environment (Table 3).

### 3.2. Correlations of sub-ME Monitoring Indicators

In order to further explore the monitoring indicators, correlation analysis was conducted among these thermal and air quality factors using Spearman coefficient (rs). Table 4 indicates that temperature was positively related to PM_10_ and TVOC in a low degree (rs = 0.329, 0.389 respectively) (*p* < 0.01). Moreover, a low positive correlation was observed between humidity and carbon oxides (i.e., CO and CO_2_) (rs = 0.328, 0.433 respectively) (*p* < 0.01). Besides, humidity was nonlinearly related to airborne bacteria (rs = 0.213) (*p* < 0.01). However, a moderate negative correlation existed between humidity and TVOC (rs =−0.568) (*p* < 0.01).

### 3.3. Comparison of sub-MEs among Station Hall, Platform, and Train Cabin

Station halls and platforms were classified in the Code for design of metro (GB50157-2013) into underground public zones, whose interior comparison regarding sub-MEs is indicated in Table 5. Lighting environments between station hall and platform were significantly different based on spatial variability. In other words, the average of illuminance in six station halls of 135.5 lx (SD = 79.5 lx) were much higher than that in six platforms (Mean = 97.3 lx, SD = 50.9 lx) (*p* < 0.05). Nevertheless, no significant nuance was monitored between station halls and platforms with respect to thermal environment and air quality.

Comparisons of indicators were then analyzed to illustrate the difference in sub-MEs between platforms and train cabins with the same depth and carrying a large flow of passengers on the daily basis (Table 6). Based on statistics, temperature was the only thermal indicator, indicating significant difference between platforms and cabins. It was detected warmer on platforms (Mean = 26.8 °C, SD = 0.6 °C) than in train cabins (Mean = 26.6 °C, SD = 0.4 °C) (*p* < 0.05), as different temperatures were set separately by air conditionings in these two locations when conducting the field sampling. Regarding air quality, carbon oxides including CO_2_ and CO on platforms of 0.044% (SD = 0.010%) and 0.3 mg m^−3^ (SD = 0.2 mg m^−3^) were around 18% and 33% heavier than those in cabins with concentrations of 0.037% (SD = 0.005%) and 0.2 mg m^−3^ (SD = 0.1 mg m^−3^), respectively (*p* < 0.05). Likewise, underground platform PM_10_ concentration of 0.185 mg m^−3^ (SD = 0.128 mg m^−3^) were significantly higher than that inside trains of 0.050 mg m^−3^ (SD = 0.017 mg m^−3^) (*p* < 0.05), and we also found the total count of airborne bacteria on platforms (Mean = 5.9 CFU per dish, SD = 5.7 CFU per dish) much more than that in cabins of carriages (Mean = 2.1 CFU per dish, SD = 1.2 CFU per dish) (*p* < 0.05). Regarding the sub-ME of lighting environment underground, it was however brighter in train cabins, with illuminance of 566.4 lx (SD = 34.9 lx), as compared with platforms whose illuminance was monitored as 97.3 lx (SD = 50.9 lx) (*p* < 0.05).

As for the sub-ME of acoustic environment on station platforms, significant difference was found between conditions with and without train running (*p* < 0.05), which illustrates that noises generated from train arriving or leaving are the primary hubbub underground. Simultaneously, on the six platforms monitored apart from DXG station, it was much noisier when trains were leaving than arriving (*p* < 0.05). Table 7 summarizes the results.

## 4. Discussion

### 4.1. Improvements in Different sub-MEs

To improve the urban subway microenvironment requires categorizing environment types indoors [39]. In this research, Sub-MEs comprise an integration of thermal environment, air quality, lighting environment, and acoustic environment. We identify causes of indicators failed to meet the national standards and establish protective strategies for betterment purpose.

Poor air circulation is the dominant reason for high RH underground regarding the thermal environment of NMLX. There are also a wide range of moisture from people, facilities, subway envelopes, above ground, etc. [39]. NRS and NFUX are the two stations near urban water areas, it is therefore easy for hot humid air from outside to flow in through the entrances into subway station halls. Meanwhile, sampling points where RH exceeds the standardized limit detected are commonly the hygroscopic sites close to washrooms, tool-rooms, or below supply-air outlets of air-conditionings. Hence, dehumidification is proposed as the priority to subway thermal environment, especially to those sensitive sites with high humidity. It occasionally happens that air velocity surpasses the standard value, attributed to settings and amounts of ventilation devices. Changes of air flow from the outside to NMLX indoors mainly depend on central air-conditionings, and their operation conditions therefore play a direct role in air velocity. This is considered as one of key indicators affecting human comfort in enclosed areas underground. It probably results in many adverse symptoms for human being with high air velocity, such as vasoconstriction, muscle and joint aches, and even diarrhea [41]. Therefore, increase in human comfort as well as decrease in health risks could be both achieved if air velocity underground is reasonably adjusted by central HVAC systems in urban sub-MEs.

Regarding air quality, PM_10_ is the major pollution indicator in YHM and WSTR station, whose entrances are adjacent to traffic intersections. PM concentrations in station halls of these two stations fluctuated more easily due to automobile gas emissions on the ground. Similar conclusion was drawn in the research of Li et al. [16] that in-train PM concentration is likely influenced by vehicle exhausts when metro lines is beside a high traffic road. Additionally, no use of air-conditionings in ventilation seasons may aggravate high PM_10_ concentrations in subway station halls and platforms to some extent. Similarly, Vânia Martins et al. demonstrated that ventilation and air conditioning systems could improve subway air quality [11]. Non-ambient sources are main significant contributors to total metro PM exposure [31,42], including emissions from rail mechanical abrasion, and dust particle resuspensions from passenger activities [5,43]. It is therefore recommended to adopt some air protective solutions in station halls or platforms, such as utilizing air sterilization apparatus, strengthening dustproof function through grid design, installing platform screen doors (PSD), and reducing combustion sources as much as possible [35]. On the contrary, PM_10_ in train cabins which are air conditioned is lighter than that in station halls and platforms—consistent with the findings of Moreno et al. [10], V. Mugica-Álvarez et al. [28] and Querol et al. [44] that the filtration of air conditioning helps in lower PM concentrations. Similarly, in-cabin PM_2.5_ exposure was also detected lighter than stations in a measurement campaign for Shanghai subway in 2015 [45]. Placement of dustbins could be another explanation for excessive PM levels, since they were found located next to all points of YHM and WSTR where PM_10_ concentrations were monitored exceeding borderlines in field sampling. As Moreno and Miguel [6] stated, platforms or even tunnels should be cleaned regularly to avoid the build-up of particulates and underground night work teams contribute greatly to minimize fugitive dusts.

Lighting environment of sub-MEs is largely associated with quantities of lamps opened. For energy saving, only half or even less the number of lamps were turned on in some station hall or platform of NMLX. The design of interchange DXG is not in line with people-oriented principles with low illuminance supplied for large flows of passengers. As a result, lighting comfort should be the basis to save electrical energy during lamp management underground. Apart from controls on the quantities of lamp opened, more intelligent designs linked to subway lighting environment could be conducted for the balance between energy saving and lighting comfort, e.g., to increase the transparency of lampshades, to choose lamps with high optical efficiency, or to apply smart building lighting systems according to real-time passenger scales.

Rolling noise commonly generates from the roughness of wheel and rail surfaces or piston effect when the subway runs [46,47]. Although the noise monitored on each platform in NMLX is lower than the maxima in standards, some inspectors complained they suffered from tinnitus or other ear problems after a long work period on subway platforms. It is confirmed the long-term exposure to noisy environment results in noise-induced hearing loss and gradually, dysfunction in human nervous system along with symptoms of daytime fatigue, hypertension, neurasthenia and so forth [48,49]. Therefore, subway operation companies should assume the responsibility to rearrange reasonable work time for the inspectors and to provide them with ear protectors and regular occupational health examinations due to their special work environment underground. Meanwhile, it is also imperative to optimize designs or increase amounts of mufflers to minimize rolling noise in subway tunnels.

### 4.2. Future Directions of sub-MEs Standards

Despite of the similarity with confined indoor microenvironment of a building, sub-ME shows quite difference with commuters exposed during their short subway trips [50]. Therefore, the elaboration of future standards or norms related to urban subway microenvironment should resort to its disparity as mentioned above. Many countries/regions have promulgated ambient air quality standards within legal framework so as to protect public health [51]. In contrast, there are few international standards systematically categorizing subway microenvironments underground. Meanwhile, by comparing the field measurements and those standardized in this research, several defects were found in the current national standards related to subway environments, such as incomplete information, antiquated versions, and poor pertinence to sub-MEs. It is therefore required to take integration, people-orientation, and precision as the principles when updating the standards or norms of sub-MEs in the future.

First, it is desirable to establish standards integrating all-round indicators. In recent years, many researches have focused on concentration distributions of fine particulate matter, viz. PM_2.5_ in underground subways [52,53,54]. However, thresholds of underground PM_2.5_ is not included in any of the existing standards. Actually, it is full of heavy metal which may increase health risks of human being [35,55]. Meanwhile, high PM_2.5_-to-PM_10_ ratios of over 50% were prior detected in many subway microenvironments [31,56,57]. These facts might undoubtedly stimulate future revisions in sub-ME-related standards. Currently, there is an exclusive requirement stated in the Ambient Air Quality Standards (GB3095-2012) that class B of air quality corresponds to average PM_2.5_ concentrations of 75 μg m^−3^ or less, while class A represents average PM_2.5_ concentrations of 35 μg m^−3^ or less [37]. Nevertheless, such a standard is more applicable to outdoor ambient air above ground and plays very limited role assessing concentration of underground PM_2.5_ in sub-MEs. The situation is reflected by our present cognitions on microenvironment indoors that substantially lags behind that outdoors [58]. In addition, there is no exposure limit of TVOC regulated in both the Hygienic standard for waiting room of public transit means (GB9672-1996) and the Code for design of metro (GB50157-2013). In fact, many VOCs originated from materials prevailing in subway air are toxic or carcinogenic and even might trigger respiratory or cardiovascular diseases [51]. Similar drawbacks could be also found regarding CO as well as total count of airborne bacteria from comparisons of relevant standards. From the above, integrating full-scaled monitoring indicators is the fundamental principle for future standards of urban subway microenvironments, especially those about air chemistry underground. Furthermore, though the existing standards have defined their own application ranges, they however failed to indicate the corresponding period of implementation. From perspectives of life cycle management and multi-objective management in urban subway systems, sub-ME is regarded as one of important subway project objectives in different stages, e.g., design, construction, and operation. Hence, it is recommended to comprise threshold of each sub-ME indicator in the whole life cycle of urban subways. Besides, other information associated with sub-MEs, such as microenvironment quality objectives, definition of indicators, and emission factors as well as emission inventories should be further covered in the future standards or norms [39].

Second, people-orientation is needed for setting standards of various micro-environmental indicators. The authorities should give priority to feelings or satisfactions of passengers in sub-MEs, as it is where larger volumes of passengers commuted in their daily life. Currently, many domestic standards of subways or indoor air quality only set extreme limits instead of values recommended based on commuter comfort. For instance, prevailing thermal environment inside trains or on platforms is of importance to the thermal comfort of passengers [59], but no corresponding levels of human thermal comfort have been included when setting values in those national standards. As for field measurements of thermal environment underground in this research, temperatures in subway station halls, on platforms, and in train cabins range in between 25.9–27.1 °C, 26.1–27.7 °C, and 26.4–27.2 °C, respectively, all of which meet the requirements according to the Hygienic standard for waiting room of public transit means (GB9672-1996) and the Code for design of metro (GB50157-2013). However, it could not be regarded as a suitable thermal condition for passengers when compared to the guidance for indoor air quality in public places of Hong Kong that a fine grade represents the temperature below 25.5 °C in public transport microenvironment [60]. Similarly, the standard ISO-7730 took human comfort into full account, which proposed three desirable classes from neutral to slightly warm in indoor thermal environment [61]. Meanwhile, as specified in ASHRAE Standard-55, limit of operative temperature rather than air temperature is worthier being introduced with respect to the feasibility of energy management in sub-MEs [62]. As for subway lighting environment underground, illuminance levels in the current Urban rail transit lighting (GB16275-2008) cannot reflect which one corresponds to the most suitable human lighting comfort either. Hence, implementation of the people-orientation principle in establishing future standards will help better understand relationships of sub-MEs and human comfort.

Third, standardization of sub-ME indicators needs to be more precise. On the one hand, it is time to update the existing standards so as to adapt to the continuous changes of urban subway environment. In China, some standards or norms regarding indoor air quality or public transport areas are too old to serve as reference for the current sub-ME situations. The Hygienic standard for waiting room of public transit means (GB9672-1996) and the Hygienic standard for bacterial total indoor air (GB/T17093-1997) were both revised in the 1990s, while the Indoor air quality standard (GB/T18883-2002) in 2002 when PM_2.5_ has not become a major health concern [63,64]. Besides, the subway systems nationwide were very young yet at that time. On the other hand, values of indicators are suggested to be comprised in an exclusive standard targeting at sub-ME situations, as currently available standards describe them separately in different versions for characterizing environment indoors. In addition, considering that the space of the station hall and platform are relatively large, it is necessary to divide them into appropriate areas according to the spatial characteristics when arranging the measurement points and setting the standard values. Through this, the accuracy is expected to be improved. In terms of temperature in subway thermal environment, it was required in the Code for design of metro (GB50157-2013) of no more than 30 °C with air conditioning opened and no more than 35 °C in emergency. However, the Indoor air quality standard (GB/T18883-2002) set thermal limits according to seasonal disparities, in which the standardized range is 22–28 °C in summer and 16–24 °C in winter. It is revealed that similar problems exist regarding other sub-ME indicators, including RH, CO_2_ concentration, CO concentration, PM_10_ concentration, and total count of airborne bacteria, which still face different thresholds in different standards or norms. 

Nowadays, field measurement campaigns are commonly deployed for micro-environmental characterization of urban subway systems [16,53,65]. Data obtained in these campaigns could then serve for the evaluation or internal correlations of sub-MEs [42,66]. The findings of this research therefore make them possible to be the foundation of sub-ME-related standards or norms in the future. While in the field of subway microenvironment research, there will be more demand for state-of-the-art measurement campaigns to improve precision of monitoring indicators. In addition, more field measurement campaigns, in a wide range of cities, are also needed. Various indicators as well as their recommended values should be further studied for updating standard(s) of urban sub-MEs in the future. Moreover, with revised/completed sub-ME-related standards or norms in hand, more standards regarding evidence-based risk assessment are expected to be formulated to further guide subway construction and operation.

## 5. Conclusions

With the aim to contribute to healthy unban subway microenvironments, field sampling experiments were conducted in typical subway station halls, subway platforms, and in-cabin trains of Nanjing Metro Line X (NMLX) in this research. The current sub-ME situations in which commuters are exposed were investigated according to comprehensive micro-environmental categories underground, including thermal environment, air quality, lighting environment, and acoustic environment. Relative humidity, air velocity, PM_10_ concentration, and illuminance were found dissatisfying the requirements in domestic standards or norms related to urban subway designs or indoor air quality. Furthermore, correlations of thermal and air quality indicators were conducted using Spearman coefficient. Comparisons of urban sub-MEs were then carried out among station hall, platform, and train cabin. There was great difference in single lighting environment between station hall and platform. Temperature on platform is the only thermal indicator - quite differs from that in train. It was detected warmer on platforms where indoor air seemed much more polluted than cabins. As for acoustic environment underground, noise of trains in and out brings the primary hubbub on platforms, and it was found much noisier when trains were leaving than arriving. Based on the measurement results of NMLX, protective strategies for improving each kind of sub-ME were proposed, with focus on the respective indicators failed to meet the standards. The research could be used as reference to take effective policy measures. Three updating principles of integration, people-orientation, and precision were further developed, and an integrated standard targeting urban sub-MEs is expected. The findings indicate the public more emphasize the issues of urban subway microenvironments, as the ambient city air has improved to some extent by phasing-out of diesel and petrol vehicles. To reduce health risks of subway microenvironments, passengers should raise self-protection awareness and conduct risk reduction behaviors, such as wearing masks. On the other hand, agency managers should reinforce the sub-ME management with more attention paid to the health of underground workers and for instance, to provide earplugs for them. This research explores broad thoughts to ensure subways operated environmentally friendly and to help promote their microenvironment quality. It provides guidance for moving towards healthy urban sub-MEs proactively on practical and regulation perspectives and in turn the sustainable operation of urban subway systems. However, there are still some limitations of this study. Correlations of passenger amount and air quality need to be further explored. Besides, as underground micro environmental problems become more serious during rush hours, sub-MEs management in these periods of time should be more emphasized in the future work.

## Figures and Tables

**Table 1:** Detailed information of monitoring sites and conditions.

Station	Category	Form
Xinghuo Road (XHR)	Suburban station	Underground two-story island
Nanjing Railway Station (NRS)	Interchange station	Underground two-story twin-island
Nanjing Forestry University Xinzhuang (NFUX)	Downtown station	Underground two-story island
Da Xing Gong (DXG)	Interchange station	Underground three-story island
Yu Hua Men (YHM)	Downtown station	Underground two-story island
West of Shengtai Road (WSTR)	Suburban station	Underground two-story island

**Table 2:** Sampling indicators and criteria of urban subway microenvironments.

Sub-ME Category	Indicator	Unit	Standard Limit
**Thermal environment**	Temperature	°C	24–28 (in summer)
	Relative humidity	%	70
	Air velocity	m s^−1^	0.5
**Air quality**	CO_2_	%	0.15
	CO	mg m^−3^	10
	PM_10_	mg m^−3^	0.25
	TVOC	mg m^−3^	0.5
	Airborne bacteria	CFU per dish	75
**Lighting environment**	Illuminance	lx	200 (station hall)
			100 (platform)
**Acoustic environment**	Noise level ^a^	dB(A)	70 (no trains running)
			80 (trains arriving)
			80 (trains leaving)

^a^ Noise level refers to equivalent consecutive sound level A (LAeq).

**Table 3:** Current sub-ME situations of station halls, platforms and train cabins in Nanjing Metro Line X (NMLX).

Station	Site	Temperature	Relative Humidity	Air Velocity	CO_2_	CO	PM_10_	TVOC	Airborne Bacteria	Illuminance	Noise Level ^a^
		°C	%	m s^−1^	%	mg m^−3^	mg m^−3^	mg m^−3^	CFU Per Dish	lx	dB(A)
**XHR ^c^**	Station hall	26.2 ± 0.1	70.0 ± 0.1	0.18 ± 0.09	0.030 ± 0.000	0.3 ± 0.2	0.068 ± 0.007	0.09 ± 0.00	2.3 ± 0.8	208.5 ± 57.7	N/A
	Platform	26.7 ± 0.1	69.6 ± 0.2	0.24 ± 0.09	0.031 ± 0.001	0.3 ± 0.2	0.075 ± 0.009	0.09 ± 0.00	2.3 ± 1.8	182.4 ± 24.5	66.1 ± 8.6
**NRS ^c^**	Station hall	26.8 ± 0.1	70.2 ± 0.1 ^b^	0.13 ± 0.07	0.061 ± 0.004	0.3 ± 0.1	0.083 ± 0.008	0.10 ± 0.00	6.3 ± 2.5	166.3 ± 63.1 ^b^	N/A
	Platform	27.4 ± 0.4	69.8 ± 0.4	0.30 ± 0.23	0.057 ± 0.004	0.2 ± 0.1	0.110 ± 0.024	0.11 ± 0.00	6.0 ± 4.3	123.5 ± 9.9	67.5 ± 7.9
**NFUX ^c^**	Station hall	26.5 ± 0.3	73.7 ± 5.5 ^b^	0.35 ± 0.18	0.051 ± 0.002	0.4 ± 0.1	0.086 ± 0.010	0.11 ± 0.00	5.0 ± 4.2	67.0 ± 24.3 ^b^	N/A
	Platform	25.7 ± 0.2	70.3 ± 0.4 ^b^	0.54 ± 0.39 ^b^	0.047 ± 0.001	0.3 ± 0.1	0.107 ± 0.033	0.11 ± 0.00	3.7 ± 2.3	54.9 ± 7.5 ^b^	68.7 ± 7.4
**DXG ^c^**	Station hall	27.7 ± 0.1	69.8 ± 0.1	0.28 ± 0.16	0.059 ± 0.007	0.3 ± 0.1	0.211 ± 0.113	0.10 ± 0.00	7.5 ± 1.5	177.2 ± 97.4 ^b^	N/A
	Platform	26.9 ± 0.2	70.5 ± 0.1 ^b^	0.08 ± 0.04	0.053 ± 0.004	0.3 ± 0.1	0.121 ± 0.008	0.11 ± 0.00	15.3 ± 7.2	103.7 ± 15.5	66.7 ± 5.7
**YHM ^c^**	Station hall	27.0 ± 0.1	70.4 ± 0.1 ^b^	0.34 ± 0.15	0.038 ± 0.001	0.9 ± 0.1	0.259 ± 0.023 ^b^	0.09 ± 0.00	4.2 ± 1.2	59.2±57.2 ^b^	N/A
	Platform	27.3 ± 0.4	70.6 ± 0.4 ^b^	0.37 ± 0.25	0.038 ± 0.002	0.6 ± 0.1	0.336 ± 0.035 ^b^	0.09 ± 0.00	4.0 ± 1.8	84.4 ± 23.0 ^b^	68.7 ± 4.0
**WSTR ^c^**	Station hall	26.0 ± 0.1	71.1 ± 0.1 ^b^	0.50 ± 0.34	0.037 ± 0.001	0.3 ± 0.1	0.338 ± 0.198 ^b^	0.09 ± 0.00	4.5 ± 3.0	134.8 ± 36.6 ^b^	N/A
	Platform	26.7 ± 0.2	70.4 ± 0.2 ^b^	0.23± 0.10	0.038 ± 0.010	0.1 ± 0.1	0.362 ± 0.113 ^b^	0.10 ± 0.00	4.3 ± 2.7	35.0 ± 3.7 ^b^	65.6 ± 10.1
**-**	Train cabin	26.6 ± 0.4	70.2 ± 0.7	0.30 ± 0.11	0.037 ± 0.005	0.2 ± 0.1	0.050 ± 0.017	0.10 ± 0.02	2.1 ± 1.2	566.4 ± 34.9	N/A

^a^ Average noise under three conditions with no trains running, pulling in and out. ^b^ Dissatisfying the standard limits in Table 2. ^c^ Abbreviations: XHR, Xinghuo Road Station; NRS, Nanjing Railway Station; NFUX, Nanjing Forestry University Xinzhuang Station; DXG, Da Xing Gong Station; YHM, Yu Hua Men Station; WSTR, West of Shengtai Road Station.

**Table 4:** Correlation of thermal and air quality environment monitoring indicators.

Monitoring Indicators	Temperature	Humidity	Air Velocity
**CO_2_**	−0.050	0.328 **	−0.059
**CO**	−0.141	0.433 **	0.011
**PM_10_**	0.329 **	−0.116	−0.118
**TVOC**	0.389 **	−0.568 **	0.058
**Airborne bacteria**	0.038	0.213 **	−0.122

** Difference is significant at the *p* < 0.01 level (2-tailed).

**Table 5:** Comparison of thermal environment, air quality, and lighting environment between station hall and platform in Nanjing Metro Line X (NMLX).

Site	Temperature	Relative Humidity	Air Velocity	CO_2_	CO	PM_10_	TVOC	Airborne Bacteria	Illuminance
	°C	%	m s^−1^	%	mg m^−3^	mg m^−3^	mg m^−3^	CFU Per Dish	lx
Station hall	26.7 ± 0.6	70.9 ± 2.5	0.30 ± 0.21	0.046 ± 0.012	0.4 ± 0.3	0.174 ± 0.135	0.10 ± 0.01	5.0 ± 2.8	135.5 ± 79.5
Platform	26.8 ± 0.6	70.2 ± 0.5	0.29 ± 0.25	0.044 ± 0.010	0.3 ± 0.2	0.185 ± 0.128	0.10 ± 0.01	5.9 ± 5.7	97.3 ± 50.9
*p*-value	0.752	0.134	0.967	0.444	0.080	0.721	0.227	0.362	0.018 *

* Difference is significant at the *p* < 0.05 level (2-tailed).

**Table 6:** Comparison of thermal environment, air quality, and lighting environment between platform and cabin in Nanjing Metro Line X (NMLX).

Site	Temperature	Relative Humidity	Air Velocity	CO_2_	CO	PM_10_	TVOC	Airborne Bacteria	Illuminance
	°C	%	m s^−1^	%	mg m^−3^	mg m^−3^	mg m^−3^	CFU Per Dish	lx
Platform	26.8 ± 0.6	70.2 ± 0.5	0.31 ± 0.24	0.044 ± 0.010	0.3 ± 0.2	0.185 ± 0.128	0.10 ± 0.01	5.9 ± 5.7	97.3 ± 50.9
Cabin	26.6 ± 0.4	70.2 ± 0.7	0.30 ± 0.11	0.037 ± 0.005	0.2 ± 0.1	0.050 ± 0.017	0.10 ± 0.02	2.1 ± 1.2	566.4 ± 34.9
*p*-value	0.020 *	0.706	0.802	0.001 *	0.045 *	0.000 *	0.616	0.000 *	0.000 *

* Difference is significant at the *p* < 0.05 level (2-tailed).

**Table 7:** Comparison of acoustic environment among six subway platforms in Nanjing Metro Line X (NMLX).

	**XHR^a^**	**NRS ^a^**	**NFUX ^a^**	**DXG ^a^**	**YHM ^a^**	**WSTR ^a^**
**Whether Or Not Train Running**						
Train running	71.9 ± 0.7	72.6 ± 1.7	72.6 ± 0.9	69.5 ± 1.8	71.2 ± 1.2	71.1 ± 1.1
No train running	54.6 ± 4.2	57.2 ± 4.3	60.8 ± 8.4	61.1 ± 6.9	63.7 ± 2.4	54.6 ± 11.2
*p*-value	0.000 *	0.000 *	0.000 *	0.004 *	0.000 *	0.001 *
**Periods**						
Train arriving	71.4 ± 0.1	71.6 ± 1.9	72.0 ± 0.7	68.2 ± 1.3	70.4±0.9	70.2 ± 0.6
Train leaving	63.5 ± 9.6	65.4 ± 8.9	67.0 ± 8.7	66.0 ± 6.9	67.9 ± 4.6	63.3 ± 11.8
*p*-value	0.002 *	0.007 *	0.020 *	0.185	0.025 *	0.016 *

^a^ Abbreviations: XHR, Xinghuo Road Station; NRS, Nanjing Railway Station; NFUX, Nanjing Forestry University Xinzhuang Station; DXG, Da Xing Gong Station; YHM, Yu Hua Men Station; WSTR, West of Shengtai Road Station. * Difference is significant at the *p* < 0.05 level (2-tailed).
